# Characteristics of bacterial pathogens associated with acute diarrhea in children under 5 years of age: a hospital-based cross-sectional study

**DOI:** 10.1186/s12879-016-1603-2

**Published:** 2016-06-07

**Authors:** Lei Tian, Xuhui Zhu, Zhongju Chen, Weiyong Liu, Song Li, Weiting Yu, Wenqian Zhang, Xu Xiang, Ziyong Sun

**Affiliations:** Department of Clinical Laboratory, Tongji Hospital, Tongji Medical College, Huazhong University of Science and Technology, Wuhan, China

**Keywords:** Acute diarrhea, Antibiotic resistance, Bacterial pathogens, Young children

## Abstract

**Background:**

Acute diarrhea is a leading cause of morbidity and mortality in children, particularly in those under the age of 5 years. *Rotavirus* is recognized as the leading cause of acute diarrhea in children, however, the contribution of bacterial pathogens as causative agents varies throughout the world. Here we report a hospital-based prospective study to analyze the characteristics of bacterial pathogens associated with acute diarrhea in children under 5 years of age.

**Methods:**

Stool samples were collected from 508 patients with acute diarrhea under 5 years of age who presented at our hospital. Nine pathogens were isolated and identified by culturing, serology or PCR, these included *Salmonella* spp., *Shigella* spp., *Vibrio cholerae*, diarrheagenic *Escherichia coli* (DEC), *Aeromonas* spp., *Plesiomonas* spp., *Vibrio parahaemolyticus*, *Campylobacter* spp. and *Yersinia enterocolitica*. Antimicrobial sensitivity tests of these pathogens were conducted. The most commonly detected pathogen, *Salmonella* spp., was further investigated by PCR and sequencing of antibiotic resistance-related genes.

**Results:**

Pathogens were identified in 20.1 % of the 508 samples. The most commonly detected pathogens were *Salmonella* spp. (8.5 %), followed by DEC (4.7 %), *Campylobacter jejuni* (3.0 %) and *Aeromonas* spp. (2.0 %). The resistance rates to ampicillin and tetracycline in *Salmonella* spp. were >60 %, but were <30 % to cephalosporins and quinolones. More than 50 % of DEC strains displayed resistance to ampicillin, cefotaxime and tetracycline, and 60 % of *C. jejuni* strains were resistant to ciprofloxacin but highly sensitive to the other antibiotics. Among 12 cephalosporin-resistant *Salmonella* isolates, TEM-1 and CTX-M-14 determinants were present in two (16.7 %) isolates. PCR screening for plasmid-mediated quinolone resistance genes revealed *gyrA* mutations in one of three highly quinolone resistant isolates.

**Conclusions:**

*Salmonella* spp., DEC, *Campylobacter* spp. and *Aeromonas* spp. were the most commonly detected bacterial pathogens in children under the age of 5 years with acute diarrhea. Our findings indicate that ampicillin and tetracycline are not suitable as first line therapeutic drugs against *Salmonella* spp. Resistance to third generation cephalosporins and quinolones was also detected. *TEM-1* and *CTX-M-14* genetic determinants, and *gyrA* mutations, were the major mechanisms associated with high levels of cephalosporin and quinolone resistance, respectively, in *Salmonella* isolates.

## Background

Diarrhea is one of the leading causes of morbidity and mortality in children under the age of 5 years worldwide, especially in developing countries such as sub-Saharan Africa and south Asia according to the report of the Global Enteric Multicenter Study (GEMS) [1]. Indeed, pediatric diarrhea accounts for >800,000 deaths per year globally (approximately 11 % of the 7.6 million estimated annual global child deaths) [2, 3]. Targeted interventions are important for reducing diarrhea-associated morbidity and mortality. The GEMS was conducted to ascertain clinical and epidemiological data of moderate-to-severe diarrhea in children aged 0–59 months [4–7]. Although China is one of the 15 high-incidence countries, unfortunately, it was not included in the GEMS study.

The main cause of acute diarrhea in children is infectious organisms, including viruses, bacteria and parasites [1, 2]. Along with improvements in living standards and health conditions, the incidence of parasite infections has decreased, with viruses and bacteria now being predominantly responsible for acute diarrhea in children [1, 2]. Human *rotavirus* is a major causative agent of diarrhea in children, especially in those <5 years of age. Most reports worldwide agree that *rotavirus* is the primary cause of acute diarrhea in children [2, 8–10]. However, the etiology of bacteria causing diarrhea appears to differ depending on geographical area. For example, a report from Spain indicated that *Campylobacter* spp*.* and *Salmonella* spp. were the primary bacterial pathogens, accounting for 22.2 % and 16.4 % of cases of acute diarrhea in children, respectively [11]. In Ecuador, *Shigella* spp*.* and *Campylobacter jejuni* were reported to be the main etiological causes of diarrhea [9]. Whereas in Turkey, *Salmonella* spp*.* (25.6 %) and *C. jejuni* (18.3 %) were the main causes of acute gastroenteritis in children [12].

China covers a large geographical area and due to the different levels of economic development between regions, significant differences exist in the causes of infectious diarrhea in children. According to a report by XinWang et al., a significant difference in the etiology of bacterial diarrhea existed between children in developing and developed regions of China [13]. The detection rate of *Shigella* spp*.* was 89 times higher in the developing region (county Sui, Henan province) than in the developed region (Beijing, the capital of China) studied in their report [13]. To date, few data have been reported on the etiology of acute diarrhea in children in central China.

**Fig. 1:**
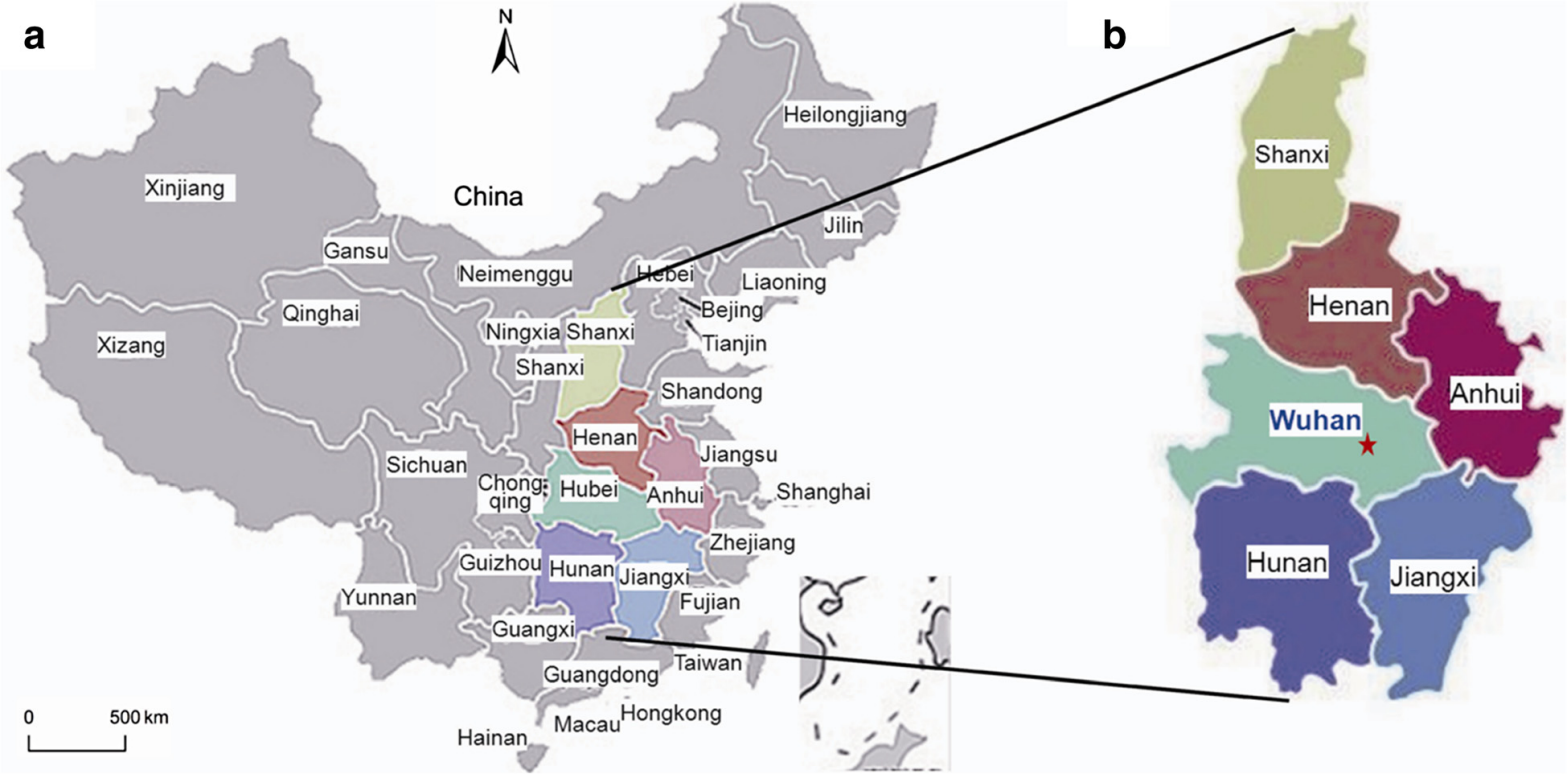
**a** the map of China. **b** the location of certral China, including six provinces, Shanxi, Henan, Anhui, Hubei, Hunan and Jiangxi

Wuhan is the capital city of Hubei province, located in the middle of central China. Tongji hospital, which has more than 4000 beds, is the largest teaching hospital in central China, located in Wuhan, and treats patients from the six surrounding provinces (Xiangxi, Henan, Hubei, Hunan, Anhui and Jiangxi). Our study analyzed fecal samples from 508 children, under 5 years of age, who presented with acute diarrhea at Tongji hospital (Fig. 1).

## Methods

### Case recruitment

Acute diarrhea was defined as three or more liquid, loose, mucus, or bloody stools within 24 h, lasting no longer than 14 days. Persistent diarrhea was defined as diarrhea that lasted for more than 14 days at presentation [14, 15]. Patients with persistent diarrhea were excluded from this study. Fever was defined as a temperature of ≥37.5 °C. If the parents or legal guardians accepted participation in the study, children under 5 years of age with acute diarrhea who were outpatients at our hospital were selected. Demographic information for each patient, including age, sex, address and clinical symptoms, was collected.

### Sample collection and microbiological methods

From May 2014 to Aug 2015, samples were collected consecutively from patients whose parents or legal guardians agreed to take part in the study. All of the parents or guardians of the patients signed the informed consent. A stool sample was collected, prior to treatment with any prescribed antibiotics. A sterilized cotton swab was dipped in the mucus, purulent or bloody part of the stool sample, then immediately placed in Cary-Blair Medium (Oxoid, United Kingdom). The samples were sent to the laboratory for immediate testing. In the laboratory, samples were cultured in different media to detect nine bacteria, these included *Salmonella* spp*., Shigella* spp*., Vibrio cholerae,* diarrheagenic *Escherichia coli* (DEC)*, Aeromonas* spp*., Plesiomonas* spp*., Vibrio parahaemolyticus, Campylobacter* spp*.* and *Yersinia enterocolitica*. Xylose lysine deoxycholate medium (XLD), MacConkey agar (Mac) and thiosulfate–citrate–bile salts–sucrose (TCBS) agar were used to isolate the pathogens conventionally (with the exception of *Campylobacter*). To further characterize *Salmonella* spp*.*, sulfa enrichment broth (SBG) (Qingdao HopeBio-Technology, China) was used to enhance growth of the bacterium. Cefoperazone deoxycholate agar (CCDA) and Skirrow’s medium were used to culture *Campylobacter* spp*.* in a microaerophilic environment at 42 °C. Phosphate buffered saline (PBS) was used for the incubation of *Yersinia enterocolitica* to increase the bacterium concentration over 10 days at 4 °C.

Strains that could not be easily identified were further investigated by manual biochemical reaction methods and/or instruments (VITEK-2 COMPACT, Biomerieux, France). Suspected *Campylobacter* colonies were subjected to PCR analysis to confirm the identification, using a method previously reported by Denis et al [16, 17].

Serotype identification was carried out by slide agglutination tests for *Salmonella* spp*., Shigella* spp*.* and *Vibrio cholerae* (using *Salmonella, Shigella* spp*.* and *V. cholerae O1, 0139* antiserum Diagnostic Antisera Kit, from Lanzhou Institute of Biological Products Co., Ltd. Lanzhou, China). Five distinct classes of diarrheagenic *Escherichia coli* (DEC) are recognized as being associated with diarrheal disease: enteropathogenic *E. coli* (EPEC)*,* Shiga toxin-producing *E. coli* (STEC)*,* enteroaggregative *E. coli* (EAEC)*,* enteroinvasive *E. coli* (EIEC) and enterotoxigenic *E. coli* (ETEC). PCR was used to distinguish these *E. coli* pathotypes by amplification of the following gene targets: typical EPEC (*eae* and *bfp*), atypical EPEC (*eae* or *bfp*), STEC (*eae* and *stx1* and/or *stx2*), ETEC (*elt* and/or *estIa* or *estIb*), EIEC (*invE* and *ipaH*) and EAEC (*aggR* and/or *pic* or *astA*) [18, 19].

### Antibiotic resistance testing

To test antibiotic resistance in *Campylobacter* spp*.*, the broth microdilution method was used with 5 % sheep blood. For all other pathogens, antimicrobial susceptibilities were determined by the agar dilution method according to the Clinical and Laboratory Standards Institute (CLSI) Guidelines, 2015 [20]. All isolates of *Salmonella* spp*.* were tested for their minimum inhibitory concentrations (MICs) of ampicillin, ampicillin-sulbactam, ceftriaxone, cefotaxime, nalidixic acid, ciprofloxacin, levofloxacin, co-trimoxazole, azithromycin, chloramphenicol and tetracycline (Oxoid); DEC were tested for ampicillin, ampicillin-sulbactam, cefotaxime, ciprofloxacin, levofloxacin, chloramphenicol, tetracycline, cefazolin, cefuroxime, imipenem, amikacin and gentamicin (Oxoid); *Campylobacter* spp*.* were tested for ciprofloxacin, azithromycin, tetracycline, erythromycin and doxycycline (Oxoid); and *Aeromonas* spp*.* were tested for cefotaxime, ciprofloxacin, levofloxacin, co-trimoxazole, chloramphenicol, tetracycline, cefazolin, cefuroxime, imipenem, amikacin and gentamicin (Oxoid). ATCC 25922, 35218, 700603 and 27853 were used as quality control strains. Antibiotic susceptibility was interpreted according to CLSI guidelines, 2015 [20].

### Molecular characterization of antibiotic resistance genes of *Salmonella* spp*.* to cephalosporins and quinolones

Those strains showing significantly decreased susceptibility to ceftriaxone and cefotaxime (MIC ≥32 mg/Ml) were further studied by PCR amplification and sequencing of the extended-spectrum β-lactamase genes (ESBLs, including *blaSHV*, *blaTEM* and *blaCTX-M*). Those isolates showing high resistance to quinolones (MIC ≥32 mg/mL to ciprofloxacin or levofloxacin) were further tested for amino acid changes in the plasmid-mediated quinolone resistance genes *gyrA* and *gyrB* by PCR and sequencing, according to the methods reported by Yenkao et al [21]. The DNA sequences were compared with sequences in the GenBank database (http://www.ncbi.nlm.nih.gov/genbank/) and the β-lactamase classification system (http://www.lahey.org/studies/) to confirm the subtypes of the β-lactamase genes.

### Statistical analysis

The chi-squared (*x*^2^) test was used to determine the statistical significance of the data by the software PASW Statistics 18.0 (IBM Corporation, New York). A *P*-value of <0.05 was considered statistically significant.

## Results

### Clinical features

During the data collection period (2014.5.1–2015.8.31), a total of 508 children aged 0–59 months who visited our hospital as outpatients were recruited to this study. Of the children, 295 (58.1 %) were male and 213 (41.9 %) were female. All of the parents or legal guardians of the patients agreed to participate in the study. A fecal specimen was collected from each patient. Blood and mucus were uncommon in the feces (1.0 % and 3.7 %, respectively). Of the 508 patients, 64.76 % (329/508) were infants (0–11 months), 22.24 % (113/508) were toddlers (12–23 months), and the other 12.99 % (66/508) were children (24–59 months). Fever and vomiting were the main symptoms accompanying acute diarrhea. Up to 57.9 % (294/508) of patients stayed in hospital for 1–3 days. There was a significant difference in the duration of diarrhea across the three age groups in the all studied pathogens (chi-squared test, *p* < 0.05), whereas no significant difference was observed in the duration of diarrhea and symptoms across the three age groups with *Salmonella* infection (chi-squared test, *p* > 0.05).

### Occurrence of pathogens

**Fig. 2:**
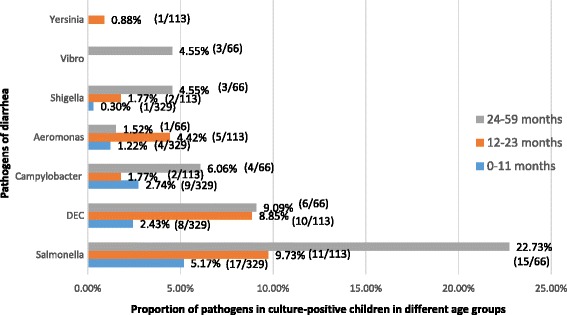
Frequency distribution of bacterial pathogens isolated from stool samples of 508 patients with acute diarrhea according to age group. The bars showed the percentage of the pathogens in culture-positive children in different age groups

In total, 102 strains of bacterial pathogens were isolated from 508 patients. Co-infection of bacteria was not detected in any of the cases. *Salmonella* spp*.* accounted for 42.2 % of the detected bacterial pathogens, followed by DEC 23.5 %, *Campylobacter* spp*.* 14.7 %, *Aeromonas* spp*.* 9.8 % and *Shigella* spp*.* 5.9 %. No *Vibrio cholerae* and *Plesiomonas* spp*.* were isolated from the 508 samples (Fig. 2). All of the *Campylobacter* spp*.* was confirmed to be *C. jejuni* by PCR. The 43 strains of *Salmonella* spp*.* detected were distributed among 10 serotypes, with *S. Typhimurium* (O4Hi) being the predominant serotype identified (18/43, 41.9 %). Among 24 strains of DEC, 11 strains of EPEC, 10 strains of STEC and three strains of EAEC were confirmed by PCR typing. Of the six strains of *Shigella* spp., four were *S. flexneri* and two were *S. sonnei* as determined by serum agglutination assays (Table 1).

**Table 1:** Serotype distribution of Salmonella, DEC, Shigella and other bacterial pathogens isolated from acute diarrheal patients

Pathogens		No. of isolates
Salmonella spp.		43/508(8.5 %)
	O4Hi	18/43(41.9 %)
	O4Hb	6/43(14.0 %)
	O9Hd	4/43(9.3 %)
	O4Hf	3/43(7.0 %)
	O4Hd	3/43 (7.0 %)
	O7Hk	3/43(7.0 %)
	O7Hf	2/43(4.7 %)
	O7Hr	2/43(4.7)
	O4Hh	1/43(2.2 %)
	O2Ha	1/43(2.2 %)
DEC		24/508(47.2 %)
	EPEC	11/24(45.8 %)
	STEC	10/24(41.7 %)
	EAEC	3/24(12.5 %)
	EIEC	0
	ETEC	0
Campylobacter spp		15/508(3.0 %)
	Campylobacter jejuni	15/15(100 %)
Aeromonas spp		10/508(19.7 %)
	Aeromonas hydrophila	8/10(80 %)
	Aeromonas sobria	1/10 (10 %)
	Aeromonas veronii	1/10(10 %)
Shigella spp.		6/508(1.2 %)
	S. flexneri	4/6(66.7 %)
	S. sonnei	2/6 (33.3 %)
Vibrio spp		3/508(0.6 %)
	Vibrio parahaemolyticus	3/3(100 %)
Yersinia spp		1/508(0.2 %)
	Yersinia enterocolitica	1/1 (100 %)
Vibro cholerae		0
Plesiomonas spp		0

### Antimicrobial resistance

**Fig. 3:**
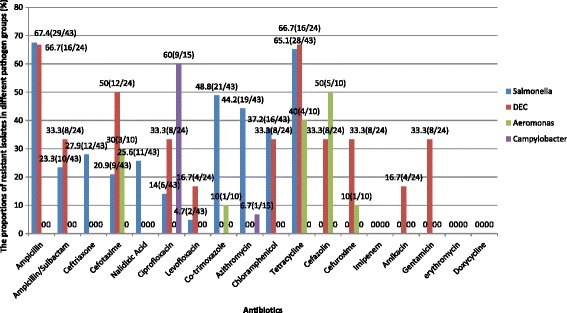
Antibiotic resistance rate of Salmonalla, DEC, Aeromonas and Campylobacter. The bars showed the percentages of resistant isolates in culture-positive children in different pathogen groups

Of the 43 strains of *Salmonella* spp*.* detected, more than 60 % were resistant to ampicillin and tetracycline. The resistance rates to co-trimoxazole and azithromycin were >40 % and to chloramphenicol was >30 %, for all other antibiotics tested the rates were <30 %. For DEC, the highest resistance rate of >60 % was detected for ampicillin and tetracycline, followed by 50 % for cefotaxime, then >30 % for ampicillin/sulbactam, ciprofloxacin, chloramphenicol, cefuroxime and gentamycin, and <30 % for levofloxacin, amikacin and imipenem. Among the 15 strains of *C. jejuni* detected, nine strains showed resistance to ciprofloxacin and one strain was resistant to azithromycin; the strains were sensitive to all other antibiotics tested. Among the 10 strains of *Aeromonas* spp*.*, five strains were resistant to cefazolin, four to tetracycline, and three to cefotaxime (Fig. 3). Drug sensitivity tests were not performed for *Shigella* spp*., Vibrio parahaemolyticus* and *Yersinia enterocolitica* because the number of strains was too small (<10) to provide interpretable results.

### Molecular analysis of plasmid-mediated quinolone resistance genes and ESBL genes

Three isolates of *Salmonella* spp*.* were found to be highly resistant to ciprofloxacin or levofloxacin (MIC ≥32 μg/mL), however, only one isolate possessed the *gyrA* gene. Twelve isolates of *Salmonella* spp. were found to be highly resistant to cefotaxime or ceftriaxone (MIC ≥32 μg/mL), three of which possessed the *TEM-1* and/or *CTX-M-14* genes (Table 2).

**Table 2:** Characteristics of 3 Salmonella spp. isolates displaying non-wild-type phenotypes high resistant to cephalosporins and quinolones

Isolate		Decreased susceptibility (MIC:ug/ml)			Gentic profile		
		Cephalosporins		Quinolones			
No	Ceftriaxone	Cefotaxime	Ciprofloxacin	Levofloxacin	Nalidixic acid	Bla gene	PMQR gene
1	4	32	64	64	64	TEM-1	-
2	1	32	64	8	64	CTX-M-14	-
3	128	32	64	1	64	TEM-1,CTX-M-14	gyr A

## Discussion

In this study, the most common pathogenic bacteria associated with acute diarrhea in children under 5 years of age were found to be *Salmonella* spp*.*, followed by DEC*, C. jejuni* and *Aeromonas* spp. These results were not consistent with the GEMS report in sub-Saharan Africa and south Asia [1]. In the case–control studies presented in the GEMS report, *Shigella* was highly associated with cases of moderate-to-severe diarrhea, EPEC showed an intermediate association, whereas *Salmonella* and *Campylobacter* were detected at similar rates in patients with or without diarrhea [1]. In a community-based study on the pathogen-specific burden of diarrhea in developing countries, which included eight study sites in South America, Africa and Asia, *Campylobacter* exhibited the highest attributable burden of diarrhea in infants (0–11 months) [22]. This may be due to distinct social, economic and environmental factors in these regions.

It was well recognized that children under 5 years of age are susceptible as a population group to *Salmonella* infection [23]. Non–typhoidal *Salmonella* (NTS) is one of the most common bacterial pathogens of foodborne infectious diseases [24]. In our study, *S. Typhimurium* was the most common serotype identified among *Salmonella* isolates, which was in agreement with previously reported studies from Niger and Guangdong Province in China [25, 26]. For the treatment of *Salmonella*, fluoroquinolones are commonly used among adults, and cephalosporins are used to treat children [27, 28]. The resistance rates to fluoroquinolones and cephalosporins among *Salmonella* have been increasing. The MIC of NTS to ciprofloxacin increased from 0.125 to 1.0 μg/ml during 2003 to 2005 in Asian countries [29]. The rate of full susceptibility of *S. typhi* to antibiotics declined from 80 % in 2002 to 28 % in 2013 in Switzerland [30]. It is therefore clear that antimicrobial resistance among *Salmonella* has become a worldwide concern [31, 32].

In our study, the resistance rates of *Salmonella* spp*.* to first line therapeutic drugs were high, i.e., 67.6 % to ampicillin, 38.2 % to chloramphenicol and 50 % to co-trimoxazole. These results were consistent with a report from Niger, where the resistant rates to amoxicillin and co-trimoxazole were >50 % for *Salmonella* spp. [26]. For quinolones and cephalosporins, we detected higher resistance rates among *Salmonella* spp*.* than those reported from other regions of the world [25, 33, 34]. This finding may be related to the wide use of cephalosporin and quinolone antibiotics in China. By molecular analysis, we identified the ESBL genes *TEM-1* and *CTX-M-14* as the genetic determinants responsible for resistance to cephalosporins, and mutations in the plasmid-mediated quinolone resistance gene *gyrA* as the major determinant of resistance to quinolone antibiotics.

In our study, more than 50 % of DEC isolates were resistant to ampicillin, cefotaxime and tetracycline, compared with <20 % for levofloxacin, amikacin and imipenem. This finding indicated that ampicillin, cefotaxime and tetracycline should not be used as first line defense against DEC infection, and that levofloxacin and amikacin might provide effective alternatives. Although most enteric infections caused by *Campylobacter* are self-limiting and do not require antibiotics, antibiotics are urgently required for prophylaxis in cases of acute diarrhea in immunocompromised patients and young children. Macrolides are recommended as the first choice therapy, with fluoroquinolones offering an effective alternative. In our study, with the exception of high ciprofloxacin resistance (60 %), *Campylobacter* isolates were sensitive to all other antibiotics tested. In a 17-year study in China, the proportion of quinolone-resistant isolates of *Campylobacter* was found to have increased markedly and reached 93.7–100 % after 2000 [35]. Similar high levels of quinolone-resistant *Campylobacter* isolates (97 % in 2008–2010) were reported in India [36]. The high prevalence of resistance to quinolones might be due to their overuse in food-producing animals [35].

This study had several limitations. It was not designed to assess attributable fractions, owing to restrictions on time and resources. In addition, the severity of diarrhea was not assessed, which may mean that milder episodes of diarrhea may have been overlooked.

## Conclusions

Our findings indicated the wide range of bacteria responsible for acute diarrhea in children under 5 years of age, including *Salmonella, Shigella,* DEC*, Campylobacter, Aeromonas, Vibrio parahaemolyticus* and *Yersinia enterocolitica*. In this particular geographical region (central China), the most commonly isolated bacterial pathogen in young children with acute diarrhea was *Salmonella* spp*.*, and *S. Typhimurium* was the most common serotype isolated. Antimicrobial susceptibility screening indicated that ampicillin and co-trimoxazole should not be used as the first line therapeutic drugs for *Salmonella* spp. In isolates of *Salmonella* spp*.* exhibiting cephalosporin and quinolone resistance, the *TEM-1* and *CTX-M-14* genes, and *gyrA* mutations, were identified as the resistance mechanisms, respectively.

## Abbreviations

CCDA, cefoperazone deoxycholate agar; *CLSI,* clinical and laboratory standards institute; *DEC*, *Diarrheogenic Escherichia coli*; *EAEC, Enteroaggregative E. coli*; *EIEC, Enteroinvasive E. coli*; *EPEC, Enteropathogenic E. Coli*; ESBL, extended-spectrum β-lactamase*; ETEC, Enterotoxigenic E. coli*; GEMS, global enteric multicenter study*;* MAC, macconkey agar*;* MIC, minimum inhibitory concentration*;* MNQR, plasmid-mediated quinolone resistance*; NTS*, *non–typhoidal Salmonella*; PCR, polymerase chain reaction*;* SBG, sulfa enrichment broth; *STEC, Shiga-toxin-producing E. coli*; TCBS, thiosulfate–citrate–bilesalts–sucrose*;* XLD, xylose lysine desoxycholate medium
